# Metabolic Characteristics and Discriminative Diagnosis of Growth Hormone Deficiency and Idiopathic Short Stature in Preadolescents and Adolescents

**DOI:** 10.3390/molecules29071661

**Published:** 2024-04-07

**Authors:** Yajie Chang, Jing Chen, Hongwei Zhu, Rong Huang, Jinxia Wu, Yanyan Lin, Quanquan Li, Guiping Shen, Jianghua Feng

**Affiliations:** 1 Department of Electronic Science, Fujian Provincial Key Laboratory of Plasma and Magnetic Resonance, Xiamen University, Xiamen 361005, China; 33320200155793@stu.xmu.edu.cn (Y.C.); 33320190153874@stu.xmu.edu.cn (J.W.); 33320200155799@stu.xmu.edu.cn (Q.L.); 2 Department of Child Health, Women and Children’s Hospital, School of Medicine, Xiamen University, Xiamen 361003, China; chenjing8469899@126.com (J.C.); huangrong83@hotmail.com (R.H.); lyy_fjxm@126.com (Y.L.); 3 Education Section and Department of Pediatrics, The First Affiliated Hospital of Bengbu Medical University, Bengbu 233004, China; zhuhongwei51136@126.com

**Keywords:** short stature, growth hormone deficiency, idiopathic short stature, metabolomics, adolescent

## Abstract

Growth hormone deficiency (GHD) and idiopathic short stature (ISS) are the most common types of short stature (SS), but little is known about their pathogenesis, and even less is known about the study of adolescent SS. In this study, nuclear magnetic resonance (NMR)-based metabolomic analysis combined with least absolute shrinkage and selection operator (LASSO) were performed to identify the biomarkers of different types of SS (including 94 preadolescent GHD (PAG), 61 preadolescent ISS (PAI), 43 adolescent GHD (ADG), and 19 adolescent ISS (ADI)), and the receiver operating characteristic curve (ROC) was further used to evaluate the predictive power of potential biomarkers. The results showed that fourteen, eleven, nine, and fifteen metabolites were identified as the potential biomarkers of PAG, PAI, ADG, and ADI compared with their corresponding controls, respectively. The disturbed metabolic pathways in preadolescent SS were mainly carbohydrate metabolism and lipid metabolism, while disorders of amino acid metabolism played an important role in adolescent SS. The combination of aspartate, ethanolamine, phosphocholine, and trimethylamine was screened out to identify PAI from PAG, and alanine, histidine, isobutyrate, methanol, and phosphocholine gave a high classification accuracy for ADI and ADC. The differences in metabolic characteristics between GHD and ISS in preadolescents and adolescents will contribute to the development of individualized clinical treatments in short stature.

## 1. Introduction

Short stature (SS) is one of the most common endocrine disorders in pediatrics [1]. It is defined as the height of a child or an adolescent below 2 standard deviations (−2 SD) or the third percentile (−1.88 SD) of the mean height of peers of the same sex. Many causes could induce child SS, such as genetic syndromes, systemic diseases, endocrine causes, and idiopathic short stature (ISS). Growth hormone deficiency (GHD) and ISS are the most common types of SS. GHD is a developmental disorder caused by partial or complete deficiency of growth hormone synthesis and secretion in the anterior pituitary gland or by receptor defects and structural abnormalities, while ISS refers to SS in children with unknown origin and normal growth hormone (GH) secretion [2]. SS not only affects the height of children, leading to abnormal bone development, but also causes nervous system dysfunction, chronic heart and lung diseases, and other diseases. Studies have also shown that children and adults with SS may have a poorer quality of life compared with people of normal stature [3]. Accurate early diagnosis and appropriate treatment are essential for effective growth improvement in adulthood [4,5]. Therefore, it is important to understand the pathogenesis of SS for diagnosis and treatment.

At present, the clinical diagnosis of SS is based on a comprehensive evaluation of medical history, the GH stimulation test, bone age X-ray film, and pituitary MRI imaging [1,6]. Because of the different treatment guidelines, the required GH dose for GHD children is different from that for ISS children. Therefore, it is necessary to distinguish GHD from ISS prior to treatment [6]. In addition, in clinical practice, the diagnostic distinction between GHD and ISS is mainly by the peak of the GH excitation test, but it has significant limitations because of the false positive rate of the stimulation test [7]. Therefore, understanding the metabolic differences between GHD and ISS can provide more effective biomarkers for early differential diagnosis of these two diseases. A study of glucose metabolism in GHD showed no significant difference between short children with peak levels of GH between 7 and 14 ng/mL and healthy children, among which the subgroup with peak GH < 10 had lower fasting insulin levels and signs of increased insulin sensitivity [8]. Serum proteomics based on a sequential windowed acquisition of all theoretical fragment ion mass spectra (SWATH-MS) technology found that a combination of apolipoprotein A-IV, complement factor H-related protein 4, and platelet basic protein showed the best classification performance between GHD children and healthy controls [9]. Our previous study based on nuclear magnetic resonance (NMR) metabolomics found that disturbed glucose metabolism and metabolism and biosynthesis of amino acids were typical metabolic features of SS caused by GHD [10]. A study on 31 children with GHD and 29 children with ISS by ultra-high-performance liquid chromatography-quadrupole/time of flight mass spectrometry (UHPLC-QTOF-MS)-based serum metabolomics found that purine metabolism, the sphingolipid signaling pathway, and sphingolipid metabolism were significantly enriched in childhood SS [11]. However, most of these studies focused on SS in preadolescents, and the metabolic information of adolescent SS was lacking. Furthermore, most studies mainly focused on the metabolic characteristics of a single disease while no comparative information on different types of SS is available. Other studies have shown that entering puberty involves important body and physiological changes when the hypothalamic–pituitary–gonadal (HPG) axis is activated. GH is responsible for growth in childhood, while the synergistic effect of sex hormones (testosterone in males and estrogens in females) and GH regulates children’s growth during puberty [12]. Therefore, an in-depth understanding of the pathogenesis of SS in preadolescents and adolescents and exploring more effective biomarkers for early diagnosis of GHD and ISS have very important clinical value and social significance.

In this study, NMR-based metabolomics combined with a machine learning method was employed to investigate the metabolomic changes caused by SS and explore the specific diagnostic biomarkers between GHD and ISS in preadolescents and adolescents. The identification of biomarkers will be beneficial in early clinical diagnosis of SS and provide a new reference for the potential individualized treatment strategy.

## 2. Results

### 2.1. Clinical Characteristics of the Study Population

**Figure 1:**
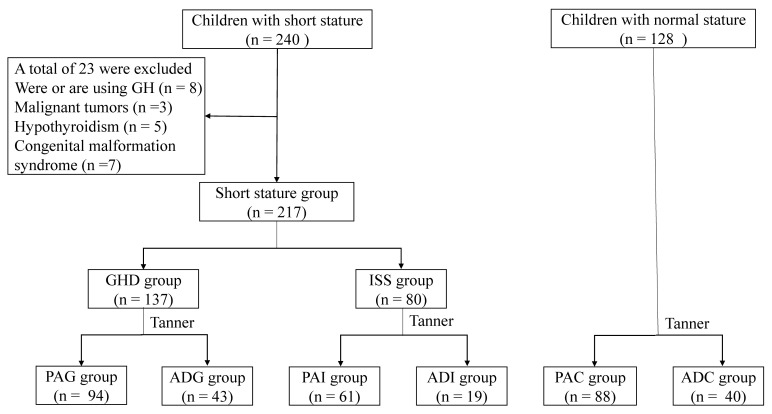
Flow chart of the participants. GH, growth hormone; GHD, growth hormone deficiency; ISS, idiopathic short stature; PAG, preadolescent GHD; ADG, adolescent GHD; ADC, adolescent control group; PAI, preadolescent ISS; ADI, adolescent ISS; PAC, preadolescent control group.

The recruitment information of the 345 children included in the present study is shown in Figure 1. The demographic and clinical characteristics of the study groups are described in Table 1. It was found that the height, weight, height standard deviation scores (Ht SDS), and alkaline phosphatase (ALP) of GHD and ISS were significantly lower than those of the corresponding healthy controls. The peak GH value of ISS was significantly higher than that of GHD. In addition, the serum levels of hematocrit (HCT), hemoglobin (HGB), and apolipoprotein A1 (APOA1) in preadolescents with SS were significantly lower than those in the control group, while free thyroxine (FT4) was higher in preadolescent GHD (PAG) and preadolescent ISS (PAI) than in the controls. Moreover, the serum levels of aspartate aminotransferase (AST) and thyroid stimulating hormone (TSH) in adolescents with SS were significantly higher than that in adolescent control children (ADC). The levels of alanine aminotransferase (ALT) and lactate dehydrogenase (LDH) in adolescents with SS were significantly higher than those in the ADC group. The *t*-test between adolescent GHD (ADG) and adolescent ISS (ADI) showed that the levels of total cholesterol (TC), low-density lipoprotein cholesterol (LDL-C), and apolipoprotein B (APOB) in ADG were higher than those in ADI, while total bilirubin (TBIL) was lower in ADG. There was no significant difference in the other clinical characteristics between groups.

### 2.2. Serum Metabolic Characteristics of Different Types of SS

**Figure 2:**
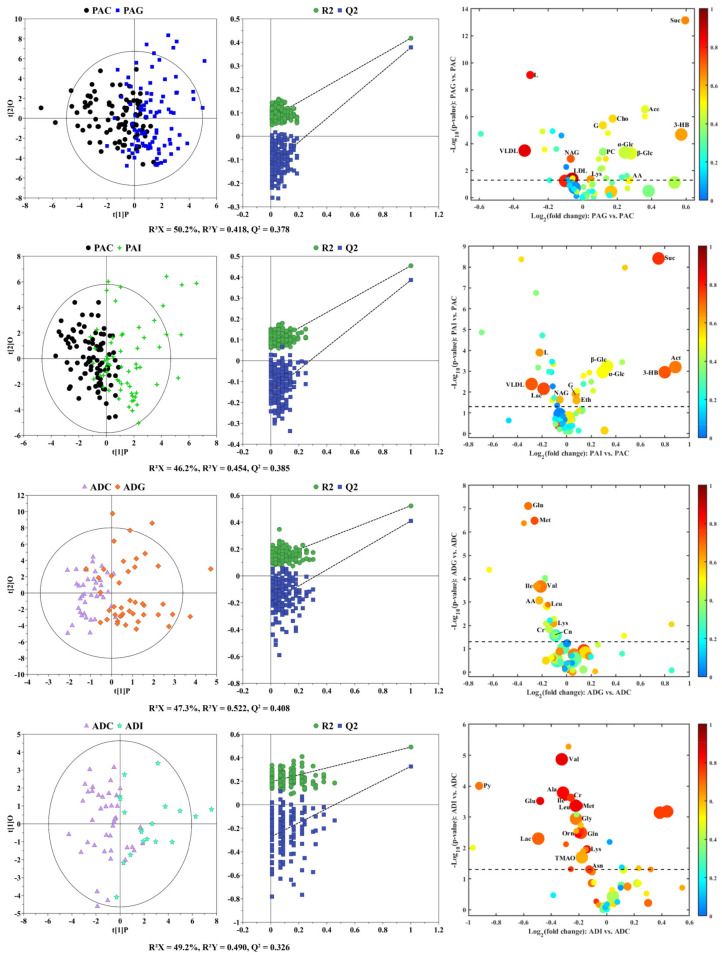
OPLS-DA score plots (left panels), the corresponding permutation test (middle panels), and volcano plots (right panels) derived from ^1^H NMR data on sera from children with SS and their corresponding controls. The marked circles in the volcano plots represent the metabolites with statistical differences between pairwise groups after correction by age and sex. Detailed information on the differential metabolites is listed in Table S1.

To understand the difference in the metabolic profiles of different types of children with SS, principal component analysis (PCA) and partial least-squares discriminant analysis (PLS-DA) score plots (Figure S1) were constructed to observe the metabolic similarity and distinction between the different groups. As shown in the PCA score plots (Figure S1(A1,B1)), no obvious separation was observed between children with SS and the controls both in preadolescence and adolescence, indicating their similar metabolic profiles. Although PLS-DA (Figure S1(A2,B2)) highlighted the inter-group differences, considerable overlap still could be observed between children with SS and their corresponding controls. To identify the potential biomarkers of different types of SS, pairwise comparisons were carried out by using orthogonal partial least-squares discriminant analysis (OPLS-DA). As shown in Figure 2, OPLS-DA highlighted the separation between the pairwise groups (Figure 2, left panels). The validity of the OPLS-DA models was verified by permutation tests (Figure 2, middle panels), where all the permutation R^2^ and Q^2^ values were lower than the original values, and the intercept values of the Q^2^ linear regression were lower than zero. According to the screening criteria and after correction by age and sex, 14, 11, 9, and 15 metabolites were identified as the potential biomarkers of PAG, PAI, ADG, and ADI (right panels of Figure 2), and their detailed information is listed in Table S1 in the Supplementary Materials. Among potential biomarkers of PAG and PAI, the contents of very low-density lipoprotein (VLDL), lipid, and N-acetyl-glycoprotein (NAG) were significantly lower than those of PAC, and the contents of glycerol, α-glucose, β-glucose, 3-hydroxybutyrate(3-HB), and succinate were significantly elevated in PAG and PAI compared with their corresponding controls. In addition, the levels of creatine, glutamine, isoleucine, leucine, lysine, methionine, and valine in ADG and ADI were lower than those in ADC.

### 2.3. Correlation Analysis between Potential Biomarkers and Clinical Index

**Figure 3:**
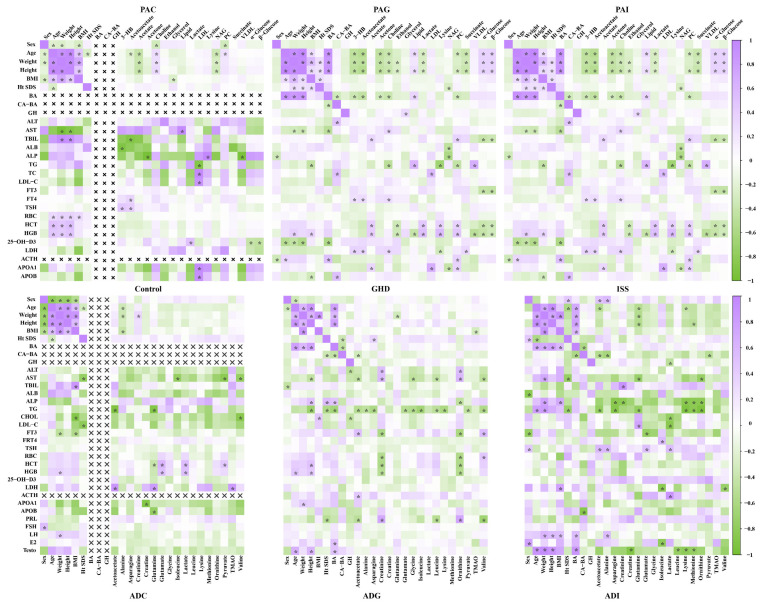
Metabolic correlation network between the potential biomarkers and clinical indices. The left panels are the control group, the middle ones are derived from the GHD group, and the right ones are derived from the ISS group. The color bars at the right represent the Pearson correlation coefficient, purple and green represent positive and negative correlation, respectively. ×, this indicator was not measured in the control groups, so correlation analysis was not warranted. *: *p* < 0.05.

To investigate the potential relationships between metabolic biomarkers and clinical indices, Pearson correlation coefficients were calculated and visualized in Figure 3. It can be clearly seen that the correlation between metabolic biomarkers and clinical index was closer in the GHD and ISS groups than in the control groups, and the correlation in preadolescents was closer than that in adolescents. In addition, the correlation between the 17 potential biomarkers of PAG and PAI and the clinical indices including age, weight, and height showed a similar trend in both the PAG and PAI groups, while a stronger correlation was observed between body mass index (BMI) and the biomarkers in the PAG group than in the PAI group. Triglyceride (TG), TC, LDL-C, and APOB were significantly associated with 3-HB, acetoacetate, acetone, choline, glycerol, lipid, lysine, NAG, and succinate in the PAG group, while HCT and HGB were significantly associated with acetate, ethanol, lactate, lysine, phosphocholine, α-glucose, β-glucose in the PAI group. Further analysis showed that ALP was positively correlated with height, Ht SDS, and bone age (BA) but negatively correlated with asparagine, creatinine, lysine, methionine, and ornithine both in the ADG and ADI groups. In the ADG group, TG had a significant negative correlation with height, Ht SDS, BA, acetoacetate, alanine, asparagine, glutamate, glycine, isoleucine, leucine, lysine, pyruvate, and valine, while in the ADI group, it was positively correlated with age and weight and negatively correlated with acetoacetate, asparagine, glutamine, lysine, methionine, and ornithine. Creatinine and ornithine showed a significant negative correlation with free triiodothyronine (FT3), red blood cell count (RBC), HCT, and HGB in the ADG group.

### 2.4. Metabolic Disturbance Induced by SS

**Figure 4:**
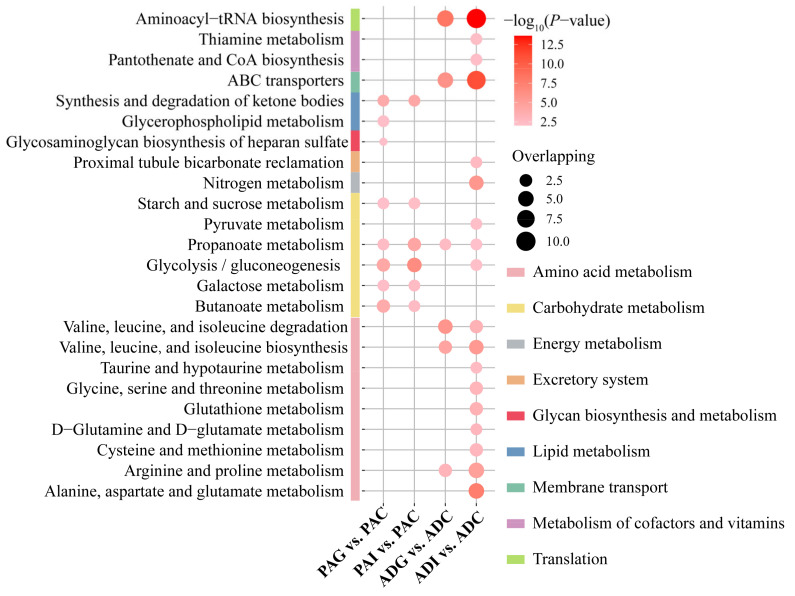
The disturbed metabolic pathways induced by PAG, PAI, ADG, and ADI. The *p*-value of the shown pathways is less than 0.01, which is filtered by ORA. PAC, preadolescent control group; PAG, preadolescent growth hormone deficiency; PAI, preadolescent idiopathic short stature; ADC, adolescent control group; ADG, adolescent growth hormone deficiency; ADI, adolescent idiopathic short stature.

**Figure 5:**
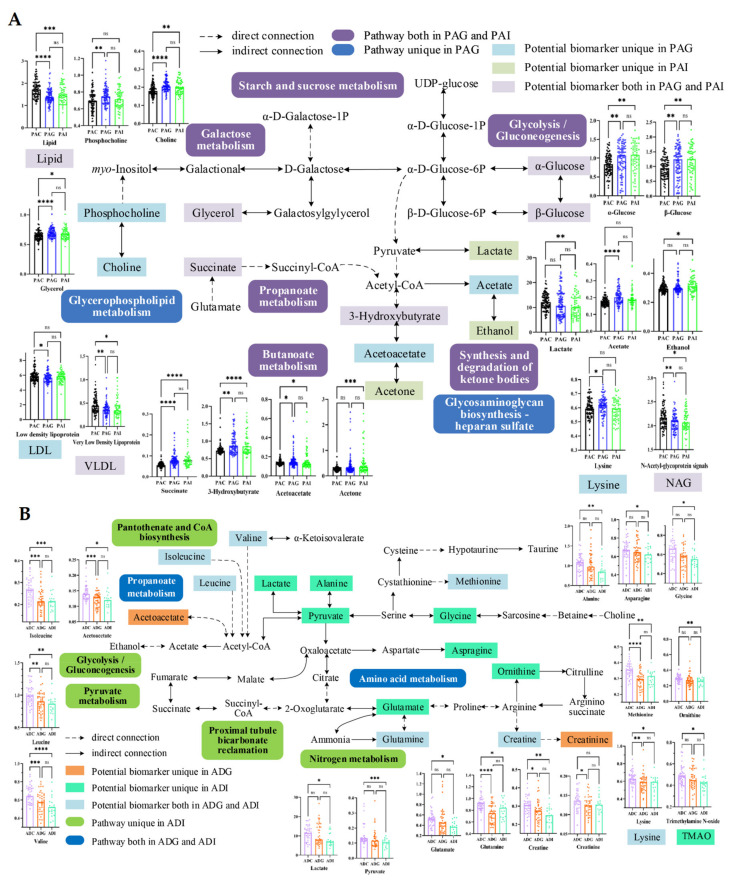
Schematic representation of the potential biomarkers and the disturbed pathways of short stature in preadolescent (**A**) and adolescent (**B**) children. *: *p* < 0.05, **: *p* < 0.01; ***: *p* < 0.001; ****: *p* < 0.0001; ns: No significance. PAC, preadolescent control group; PAG, preadolescent growth hormone deficiency; PAI, preadolescent idiopathic short stature; ADC, adolescent control group; ADG, adolescent growth hormone deficiency; ADI, adolescent idiopathic short stature.

To further understand the metabolic disorders in children with SS, pathway over-representation analysis (ORA) of potential biomarkers was performed via the online website MBROLE 2.0. Based on the criterion of *p* < 0.01, 8, 6, 6, and 18 disturbed metabolic pathways were screened out from the PAG, PAI, ADG, and ADI groups, respectively (Figure 4). According to the metabolic pathway analysis in Figure 5, carbohydrate metabolism and lipid metabolism play key roles in preadolescent SS (Figure 5A), while amino acid metabolism is significantly disordered in adolescent SS (Figure 5B). However, butanoate metabolism, galactose metabolism, glycolysis/gluconeogenesis, starch and sucrose metabolism, propanoate metabolism, and the synthesis and degradation of ketone bodies are all disturbed in the PAG and PAI groups, while disordered glycerophospholipid metabolism and glycosaminoglycan biosynthesis of heparan sulfate are observed only in the PAG group. Six disorder metabolic pathways enriched in the ADG group were also identified in the ADI group, including valine, leucine, and isoleucine degradation and biosynthesis, arginine and proline metabolism, propanoate metabolism, ABC transporters, and aminoacyl-tRNA biosynthesis. In addition, 12 disturbed metabolic pathways, including six amino acid metabolism pathways, two carbohydrate metabolism pathways, nitrogen metabolism, proximal tubule bicarbonate reclamation, thiamine metabolism, and pantothenate and CoA biosynthesis, were found uniquely in the ADI group.

### 2.5. Construction of Diagnostic Models for GHD and ISS

**Figure 6:**
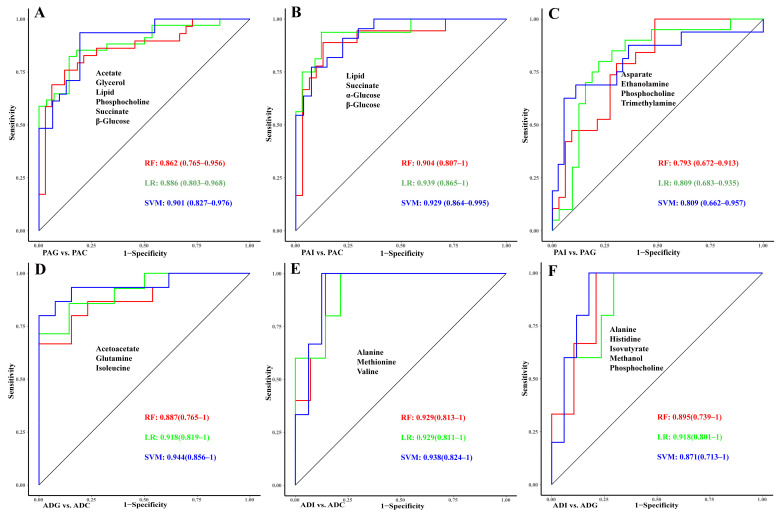
Evaluation of the diagnostic models of children with SS. The ROC curve of diagnostic models constructed by LASSO regression in PAG vs. PAC (**A**), PAI vs. PAC (**B**), PAI vs. PAG (**C**), ADG vs. ADC (**D**), ADI vs. ADC (**E**), and ADI vs. ADG (**F**). Red line, RF; green line, LR; blue line, SVM. ROC, receiver operating characteristic; LASSO, least absolute shrinkage and selection operator; RF, random forests; LR, logistic regression; SVM, support vector machine.

To determine the contribution of the metabolites to the classification of SS, least absolute shrinkage and selection operator (LASSO) regression analysis and receiver operating characteristic curve (ROC) analyses were performed based on the identified potential biomarkers (Figure 6). For the differential diagnosis between PAI and PAG and ADI and ADG, seven and seven metabolites with *p* < 0.05 were respectively included in the LASSO regression analysis (Figure 6C,F). Finally, acetate, glycerol, lipid, phosphocholine, succinate, and β-glucose were selected and combined to construct the diagnostic model for PAG from PAC, and the combination of these biomarkers showed high predictive ability with an area under the curve (AUC) of 0.901 (Figure 6A). Lipid, succinate, α-glucose, and β-glucose could be used to distinguish PAI from the controls with an AUC of 0.939 (Figure 6B). As shown in Figure 6C, four (aspartate, ethanolamine, phosphocholine, and trimethylamine) of the seven metabolites were selected as the diagnostic markers between PAI and PAG, and the ROC curves underscored the high performance with an AUC of 0.809. Similarly, the AUCs of the combination of acetoacetate, glutamine, and isoleucine are all above 0.88 (Figure 6D), suggesting that these metabolites could effectively identify ADG from ADC and be considered as the diagnostic markers of ADG. Moreover, the combination of alanine, methionine, and valine has high classification accuracy for ADI and ADC with AUC > 0.9 for all three models (Figure 6E). In addition, the AUC of the model with a combination of alanine, histidine, isobutyrate, methanol, and phosphocholine reached 0.918 (Figure 6F), which could significantly distinguish between ADI and ADG.

## 3. Discussion

The disturbed metabolic pathways in preadolescent SS were mainly involved in carbohydrate metabolism and lipid metabolism, and the increased levels of 3-HB, acetate, acetoacetate, succinate, choline, phosphocholine, glycerol, and α- and β-glucose were the keys to the disordered metabolic network.

In general, the increased levels of acetate, α- and β-glucose, and glycerol and the decreased levels of lactate clearly indicated disturbed glycolysis/gluconeogenesis, galactose metabolism, and starch and sucrose metabolism in SS. Changes in glucose levels in the body can reflect changes in energy metabolism. Under normal conditions, the body converts glycogen into glucose to ensure energy supply. The negative correlation between glycerol and TG, TC, and LDL-C in PAG and the negative correlation between α- and β-glucose with TBIL, FT3, HCT, and HGB confirmed such results. In addition, GH, as one of the insulin-resistance hormones, can increase blood glucose by inhibiting the use of glucose in muscle and adipose tissue and promoting gluconeogenesis and glycogen decomposition in the liver [13]. Therefore, GH deficiency may be associated with reduced insulin sensitivity and insulin resistance [14]. Elevated serum α- and β-glucose in PAG may indicate that GHD induced insulin resistance with reduced peripheral insulin sensitivity.

Previous studies have shown that ketone bodies (including 3-HB, acetoacetate, and acetone) are intermediate metabolites in the process of lipid oxidation metabolism and play imperative roles in mammalian cell metabolism, homeostasis, and signaling, serving as energy substances for extrahepatic tissues in the absence of glucose [15]. It has also been found that ketone bodies can regulate osteoblast function bidirectionally, suggesting that ketone bodies are an important endogenous factor in regulating bone metabolism under physiological and pathological conditions [16]. The increase in 3-HB, acetoacetate, and acetone in children with SS will affect bone metabolism, and thus further affect height. Elevated choline and phosphocholine-induced disruption of glycerophospholipid metabolism is a specific metabolic pathway that distinguishes PAG from PAI. Choline is an essential nutrient for normal growth, development, and maintenance of various functions in the human body and plays pivotal roles in membrane formation, lipid metabolism, liver and kidney function, and nerve conduction [17]. It also acts as a methyl donor and is oxidized to form betaine, and then it is converted into trimethylamine by intestinal microorganisms and oxidized to trimethylamine N-oxide (TMAO). Many studies have shown that the concentrations of TMAO, choline, and betaine in serum are closely related to the occurrence of cardiovascular diseases such as stroke and myocardial infarction [18]. Elevated serum choline may stimulate the synthesis of excessive acetylcholine by liver cells, leading to liver cell damage. The elevation of serum choline and phosphocholine in PAG may reflect liver damage in preadolescent GHD, as evidenced by elevated ALT and AST. Meanwhile, ketone bodies and choline were positively correlated with AST and TG, while TC and LDL-C were negatively correlated in PAG, indicating that disorders in ketone body metabolism and glycerophospholipid metabolism can induce simultaneously dyslipidemia.

As it is well known, amino acids play a crucial role in various biological processes, including protein synthesis, nutrient transport, and energy production. In children, amino acids are particularly important for growth and development because of their involvement in building and repairing tissues, as well as supporting the immune system and cognitive function. Our results also indicate that disorders in amino acid metabolism occupy an important position in the disturbed metabolic network of adolescent SS (Figure 5B).

Valine, leucine, and isoleucine, collectively referred to as branched-chain amino acids (BCAAs), are essential amino acids acquired solely from the diet. In this study, the decreases in BCAAs imply the disruption of valine, leucine, and isoleucine metabolism in adolescent GHD and ISS. BCAAs are not only the cornerstone of protein synthesis, but they also play important physiological roles in regulating the metabolism of glucose and lipids, intestinal health, and immunity [19]. BCAAs, especially leucine, promote the synthesis of protein in the liver and other tissues by activating the mammalian target of rapamycin (mTOR) signaling pathway [20,21]. The activation of the mTOR pathway is essential for cell growth and proliferation [22], and it can also regulate the speed of chondrogenesis and endochondral growth [23]. Therefore, a reduction in BCAAs may affect growth velocity via the mTOR pathway. Supplementation of BCAAs may improve muscle protein metabolism, body maintenance, and aerobic exercise [24,25]; therefore, the optimization of dietary BCAAs will be beneficial in improving the height of children with SS.

As the most abundant nonessential amino acid in the metabolic cycle, glutamine has multiple effects on human growth and development, including promoting cell proliferation and differentiation, improving the immune function of the body, and protecting the intestinal barrier capacity [26]. Recent evidence indicates that glutamine also plays an important role in the regulation of bone homeostasis in bone cells [27,28]. Studies on growth plate chondrocytes have shown that glutamine regulates the expression of chondrogenic genes through glutamate dehydrogenase-dependent acetyl CoA synthesis [29]. The downregulation of serum glutamine levels in adolescents with SS suggests that SS could affect bone growth and consequently lead to a lower stature.

Furthermore, lysine is one of the essential amino acids in the human body, which can promote human development and enhance immune function. Only supplemented with sufficient lysine, the body can effectively absorb and utilize protein, maintain a balanced nutrition, and promote healthy growth and development [30]. If lysine is deficient, children may experience decreased appetite, leading to negative consequences such as stunted growth, anemia, decreased resistance, and susceptibility to illness. This is consistent with the findings of this study regarding lower levels of lysine in adolescent GHD and ISS, indicating that lysine deficiency may be one of the reasons for short stature. Studies on height improvement in children in China and Pakistan have shown a significantly greater gain in the height of children receiving lysine-fortified wheat flour [31,32]. Therefore, lysine supplementation can promote growth and improve height by increasing appetite for adolescent GHD and ISS.

The levels of other metabolites, such as alanine, asparagine, glutamate, glycine, lactate, ornithine, pyruvate, and TMAO, had unique decreases in ADI, indicating that adolescent ISS involved more metabolic disorders. Among them, alanine is a nonessential amino acid made in the body from the conversion of pyruvate or the breakdown of DNA. It is highly concentrated in muscle, functioning as a major energy source [33]. Serum alanine may decrease with low BCAAs, which might be related to muscle metabolism [34]. Asparagine functions in regulating gene expression and promoting the normal operation of the immune system [35]. Glutamate plays a crucial role in nutrition, metabolism, and signaling, and is also the main excitatory neurotransmitter [36]. Studies have shown that glutamate plays an important role in the differentiation and function of osteoblasts and osteoclasts in vitro, and it is also crucial for bone growth and reconstruction [37]. Glycine plays a vital role as an antioxidant, providing energy and helping to regulate the nervous system. It also helps maintain the acid–base balance of metabolism and supports immune function in the human body. Maintaining the balance of serine and glycine is crucial for the proper proliferation of primary muscle progenitor cells in humans and the efficient regeneration of skeletal muscle [38]. In addition, ornithine is a non-essential amino acid that mainly functions in the urea cycle and liver detoxification process through the serotonin pathway [35]. The decrease in these metabolites indicates that the metabolic disorders in adolescents with ISS were more complex than in GHD.

As shown in Figure 6, the combination of aspartate, ethanolamine, phosphocholine, and trimethylamine provides the highest predictive ability for PAI from PAG. The levels of these metabolites were all higher in PAG than in PAI. Ethanolamine, as an intermediate of glycerophospholipid metabolism, further proves that the difference between preadolescent GHD and ISS may lie in lipid metabolism. GH is involved in lipid metabolism by binding to receptors on the surface of target cells [39]. Therefore, the deficiency of GH in children with PGA may lead to disturbances in lipid metabolism. The binding of GH to its receptors can activate multiple signaling pathways, and the interactions between these pathways have been shown to inhibit lipid synthesis and reduce lipid metabolism. As a result, GH supplementation through recombinant growth hormone(rhGH) injections may improve the height of children with PAG by improving lipid metabolism.

Furthermore, the combination of alanine, histidine, isobutyrate, methanol, and phosphocholine could be selected as the diagnostic model to distinguish ADG from ADI with the smallest AUC of 0.8 in ROC curves. Except for methanol, the concentrations of the other four metabolites were higher in ADG than in ADI. Phosphocholine was also identified as a biomarker for distinguishing between PAG and PAI.

## 4. Materials and Methods

### 4.1. Children Recruitment and Inclusion

This study was approved by the Human Ethics Committees of Women and Children’s Hospital, Xiamen University (KY-2016002). All procedures were in accordance with the 1964 Declaration of Helsinki and its later amendments or comparable ethical standards. All participants were recruited from Women and Children’s Hospital, Xiamen University, and the First Affiliated Hospital of Bengbu Medical University. The criteria for inclusion were as follows: (i) children whose height was below −1.88 SD for their age and gender; (ii) children with subnormal growth velocity lasting no more than six months; (iii) children who underwent a GH stimulation test; and (iv) children whose pituitary gland MRI showed no abnormalities. The exclusion criteria comprised the following: (i) children who were currently using GH or had used it previously; (ii) children with intracranial abnormalities, including malignant tumors, hypothalamic hamartoma, or Arnold Chiari malformation; (iii) children with chronic liver and kidney diseases, skeletal diseases, congenital heart disease, thyroid hormone axis abnormalities, or chromosomal abnormalities; and (iv) children whose MRI scans showed artifacts or could not be clearly outlined in the sagittal plane.

Children with GHD were these with serum peak GH concentration < 10 ng/mL on more than one occasion. On the other hand, ISS was classified as a serum peak GH concentration ≥ 10 ng/mL with provocation. According to Tanner, children with SS were classified into the following two categories: preadolescent and adolescent.

### 4.2. GH Stimulation Tests

To conduct GH stimulation tests, the children were required to fast after 8 p.m. The next morning, blood samples were collected from the median cubital vein in the arm for basic GH tests. Arginine hydrochloride in 200 mL of saline was administered intravenously to children within 30 min at a dosage of 0.5 g/kg body weight. If the GH peak of the stimulation test was <10 ng/mL, a second stimulation test was conducted by oral L-dopa at a dose of 10 mg/kg. Serum GH levels were measured at 30, 60, 90, and 120 min after administration by collecting blood samples from the other arm.

The recruitment information of the 345 children included in the present study is shown in Figure 1. Based on the etiology of SS and adolescent stage, 217 children with SS were classified into the following four groups: 94 preadolescent GHD (PAG), 61 preadolescent ISS (PAI), 43 adolescent GHD (ADG), and 19 adolescent ISS (ADI). In addition, 128 healthy children, including 88 preadolescent children (PAC) and 40 adolescent children (ADC) were recruited as the control groups based on age and gender matching.

### 4.3. Sample Collection and Preparation and ^1^H NMR Spectroscopy

Serum samples were collected in the morning after fasting overnight, left to stand for 30 min, and then centrifuged at 1000× *g* for 10 min at 4 °C. The serum supernatant was transferred to a fresh centrifuge tube and kept at −80 °C until analysis. For ^1^H NMR spectral acquisition, a serum sample (400 μL) was combined with a phosphate buffer solution (200 μL) containing 0.9% saline and D2O (99.9%, pH 7.4, 60 mM). The mixture was then centrifuged at 13,000× *g* for 10 min at 4 °C, and then the supernatant (550 μL) was extracted and carefully transferred into a 5 mm NMR tube (ST500, NORELL, Inc., Morganton, NC, USA) for further analysis.

The serum samples were subjected to ^1^H NMR analysis using a 600 MHz Bruker Advance spectrometer (Bruker Corporation, Karlsruhe, Germany) at 600.13 MHz and 298 K. To suppress signals from macromolecules and other molecules with constrained molecular motion, a typical Carr–Purcell–Meiboom–Gill (CPMG, [RD-90°-(τ-180°-τ)n-ACQ]) pulse sequence with a spectral width of 12 KHz was utilized. The acquisition time was 1.36 s, the spin-echo loop time (2nτ) was set as 70 ms, and the relaxation delay was set at 4.0 s. A total of 64 scans were collected, resulting in 16 K sampling points.

### 4.4. NMR Spectral Processing

MestReNova software (version 14.1.1; Mestrelab Research, Santiago de Compostela, Galicia, Spain) was used to process the NMR spectra. Prior to processing, all the free induction delays (FIDs) were zero-filled to 64 K data points and processed using an exponential function with a line-broadening factor of 1.0 Hz. Following Fourier transformation, all spectra underwent phase and baseline correction and were calibrated to the double peaks of endogenous lactate at δ1.33. Residual water signals (δ4.67–5.20) and urea signals (δ5.50–6.08) were eliminated, and spectral regions (δ0.6–8.6) were segmented into discrete regions of 0.002 ppm and integrated to obtain the two-dimensional data matrix, which was then normalized to its total integrated area.

### 4.5. Data Analysis

Statistical analyses of demographic data and clinical variables were processed using IBM SPSS Statistics (version 26, IBM Corporation, Armonk, NY, USA). Categorical data were presented as the number and percentage, and statistical comparison between groups was carried out using the Chi-Squared test. Continuous variables were expressed as the mean and standard deviation (SD), and Student’s *t*-test and non-parametric Mann–Whitney U test were conducted to explore the differences between pair-wise groups. One-way ANOVA was used for multiple-group comparison analysis.

The NMR data were processed using SIMCA software (version 14.1, Umetrics AB, Umea, Sweden). Before multivariate statistical analysis, the NMR dataset was Pareto-scaled. PCA and PLS-DA were conducted for an overview of different groups of preadolescents and adolescents. To determine the differences between children with SS and their corresponding healthy controls, OPLS-DA was performed, and 10-fold cross-validation ANOVA and 200 permutation tests were conducted to evaluate the over-fitting risk of the model. The variable importance in the projects (VIPs) and correlation coefficients (Cor) of the OPLS-DA model were used for the identification of differential metabolites.

Based on Chenomx NMR Suite 8.1 (Chenomx Inc., EDBonton, AB, Canada), the NMR peaks in the serum spectra were assigned to individual metabolites and confirmed by the public Human Metabolome Database (HMDB) (http://www.hmdb.ca (accessed on 8 February 2023)). A total of 59 metabolites were identified from the serum spectra of the children with SS and the control children (Figure S2 and Table S2). The assigned metabolites’ relative concentrations and fold change (FC) in the concentration between the pair-wise groups were calculated based on the integral of the corresponding spectral range. The enhanced four-dimensional volcano plots were displayed based on the FC, VIP, Cor, and *p*-value of each metabolite. The metabolites with *p* < 0.05 after age and gender correction by linear regression, |r| > 0.300 (|r| > 0.600 for ADI vs. ADC), and VIP values at the top 10% were identified as potential biomarkers. Therefore, those metabolites with an adjusted *p*-value < 0.05 were considered as potential biomarkers. Correlation (Pearson’s correlation) analyses between potential biomarkers and clinical parameters were further performed using the R (version 4.3.1) package ‘corrplot’ (https://cran.r-project.org/web/packages/corrplot/vignettes/corrplot-intro.html (accessed on 12 February 2023)).

The ORA of metabolites was conducted by MBROLE 2.0 [40] (http://csbg.cnb.csic.es/mbrole2 (accessed on 15 February 2023)), and a metabolic pathway with *p* < 0.01 was considered significantly changed. In addition, the comprehensive metabolic network was mapped with differential metabolites using the Kyoto Encyclopedia of Genes and Genomes (KEGG) (http://www.genome.ad.jp/kegg/ (accessed on 20 February 2023)) and HMDB.

### 4.6. Diagnostic Models Construction

The LASSO algorithm in the lars R package was performed to extract potential biomarkers with higher model contribution values. For pairwise comparisons of PAI vs. PAG and ADI vs. ADG, all metabolites with *p* < 0.05 (*t*-test and Mann–Whitney U test) were included in the LASSO regression analysis. The diagnostic models were constructed based on the selected biomarkers, and the predictive power was evaluated by random forests (RFs), logistic regression (LR), and support vector machines (SVMs) in the randomForest, Stats, and e1071 R packages. The ROC curve, AUC, and confidence interval (CI) of each model were carried out by the pROC R package.

## 5. Conclusions

In summary, our study indicated that carbohydrate metabolism and lipid metabolism play key roles in preadolescent SS, while amino acid metabolism pathways were significantly disordered in adolescent SS. The combination of aspartate, ethanolamine, phosphocholine, and trimethylamine exhibits the strongest predictive ability for PAI from PAG. Additionally, alanine, histidine, isobutyrate, methanol, and phosphocholine were carefully selected and integrated into a diagnostic model to effectively distinguish between ADI and ADG. Our research demonstrated that an NMR-based metabolomics strategy could provide a new perspective for constructing diagnostic models and understanding the pathogenic pathways of multi-diseases.

## Figures and Tables

**Table 1:** Demographic data and clinical characteristics of the study cohort.

	PAC ^a^ (*n* = 88)	PAG ^b^ (*n* = 94)	PAI ^c^ (*n* = 61)	*P* ^d^	ADC ^e^ (*n* = 40)	ADG ^f^ (*n* = 43)	ADI ^g^ (*n* = 19)	*P* ^h^
Sex (male)	55(62.5%)	61(64.9%)	31(50.8%)	0.192	21(52.5%)	28(65.1%)	16(84.2%)	0.059
Age (year)	7.06(2.57)	7.38(2.18)	7.41(2.56)	0.590	11.63(1.66)	12(1.51)	12.83(1.74) *	0.032
Weight (kg)	22.75(7.46) **	19.6(5.76)	19.26(5.34) **	0.001	40.16(8.94) ***	32.13(8.13)	32.92(8.16) **	0.000
Height (cm)	119.8(14.8) ***	111.9(11.8)	112.9(13.9) **	0.000	151.6(10.2) ***	134.3(8.1) *	139.8(8.5) ***	0.000
BMI (kg/m^2^)	15.42(1.5)	15.31(2.12) *	14.78(1.13) **	0.076	17.24(1.99)	17.34(4.36) *	16.66(2.87)	0.751
Ht SDS	−0.05(0.89) ***	−2.54(0.62) **	−2.29(0.42) ***	0.000	0.19(0.71) ***	−2.58(0.63)	−2.55(0.55) ***	0.000
BA	-	5.75(2.35)	6.24(2.56)	-	-	10.97(1.68)	11.56(1.93)	-
CA-BA	-	1.97(1.02)	1.71(1.1)	-	-	1.15(1.04)	1.51(1.36)	-
Peak GH (ng/mL)	-	6.64(2.15) ***	14.34(4.35)	-	-	6.34(2.12) ***	18.27(6.05)	-
Liver function								
ALT (U/L)	14(3)	16(5)	16(4)	0.619	13 (2) **	17 (6)	19(6) *	0.091
AST (U/L)	26(4)	33(7)	34(7) *	0.080	22(4) *	27 (6)	32 (9) *	0.013
TBIL (μmol/L)	14.1(3.9)	11.7(4.9)	11.9(5.5)	0.593	14.6(5.2)	12.3(4.3) *	17.0(7.1)	0.096
ALB (g/L)	45.4(2.8) *	43.4(2.2)	43.3(2.6)	0.156	44.5(1.7)	44.4(2.4)	43.8(1.2)	0.685
ALP (U/L)	287(87)	192(42)	203(47) **	0.000	295(102) *	210(65)	227(81)	0.041
Blood lipid								
TG (mmol/L)	0.78(0.26) *	0.59(0.17)	0.62(0.23)	0.093	0.80(0.26)	0.79(0.22)	0.85(0.36)	0.840
TC (mmol/L)	5.01(1.50)	4.69(0.96)	4.52(0.92)	0.481	4.21(0.59)	4.91(1.24) *	4.12(0.44)	0.112
LDL-C (mmol/L)	2.52(0.91)	2.66(0.75)	2.61(0.74)	0.895	2.19(0.36)	2.75(0.94) *	1.98(0.43)	0.043
Thyroid								
FT3 (pg/mL)	4.30(0.56)	4.44(0.54)	4.47(0.47)	0.408	4.32(1.15)	4.4(0.77)	4.58(0.29)	0.794
FT4 (ng/dL)	0.95(0.12) *	1.02(0.15) *	1.09(0.18) ***	0.000	0.91(0.11)	1.52(3.21)	2.76(5.03)	0.107
TSH (μIU/L)	2.389(1.037)	3.039(2.036) **	2.311(1.038)	0.012	2.029(0.836) **	2.971(1.457)	2.546(1.192)	0.003
Blood routine								
RBC (×10^12^/L)	4.66(0.33)	4.63(0.44)	4.48(0.28) **	0.052	4.71(0.26)	4.65(0.31)	4.75(0.34)	0.675
HCT (g/L)	37.9(2.3) *	37.0(2.1)	36.9(1.6) **	0.018	40.3(2.3)	39.3(2.5)	38.5(3.5)	0.209
HGB (g/L)	129.1(9.1) *	125.3(8.0)	125.2(7.0) *	0.013	137.1(7.5)	134.6(10.1)	128.3(11.1) *	0.082
Others								
25-OH-D3 (nmol/L)	80.85(24.30)	92.17(32.45)	86.59(26.96)	0.231	76.21(25.29)	73.40(21.49)	70.34(14.01)	0.808
LDH(U/L)	257(39)	278(69)	287(76)	0.602	196 (19) **	229 (39)	231(248) **	0.075
ACTH (pmol/L)	-	5.58(3.19)	4.62(3.20)	-	-	7.39(4.24)	6.62(2.89)	-
APOA1 (g/L)	1.70(0.27)	1.54(0.19) *	1.46(0.22) *	0.021	1.42(0.12)	1.54(0.21)	1.45(0.09)	0.197
APOB (g/L)	0.68(0.29)	0.72(0.19)	0.71(0.18)	0.849	0.59(0.1)	0.74(0.25) *	0.55(0.10)	0.056
PRL (ng/mL)	-	-	-	-	8.14(4.46)	10.43(7.88)	7.39(2.08)	0.337
FSH (mIU/mL)	-	-			4.33(2.34)	9.29(30.83)	4.65(2.48)	0.621
LH (mIU/mL)	-	-	-	-	1.67(1.32)	2.25(4.03)	2.15(1.52)	0.763
E2 (pg/mL)	-	-	-	-	32(13)	25(19)	36 (21)	0.195
Testo (ng/mL)	-	-	-	-	0.99(1.56)	0.93(1.16)	1.9(2.1)	0.327

Abbreviations: PAC, preadolescent control group; PAG, preadolescent growth hormone deficiency; PAI, preadolescent idiopathic short stature; ADC, adolescent control group; ADG, adolescent growth hormone deficiency; ADI, adolescent idiopathic short stature; BMI, body mass index; Ht SDS, height standard deviation score; BA, bone age; CA-BA, bone age retardation; GH, peak growth hormone; ALT, alanine aminotransferase; AST, aspartate aminotransferase; TBIL, total bilirubin; ALB, albumin; ALP, alkaline phosphatase; TG, triglyceride; TC, total cholesterol; LDL-C, low-density lipoprotein cholesterol; FT3, triiodothyronine; FT4, free thyroxine; TSH, thyroid stimulating hormone; RBC, red blood cell count; HCT, hematocrit; HGB, hemoglobin; 25-OH-D3, 25-dihydroxyvitamin D3; LDH, lactate dehydrogenase; ACTH, adrenocorticotropic hormone; APOA1, apolipoprotein A1; APOB, apolipoprotein B; PRL, Prolactin; FSH, follicular stimulating hormone; LH, luteinizing hormone; E2, estradiol; Testo, testosterone. Values are presented as means ± SDs or n (%). *: *p* < 0.05, **: *p* < 0.01; ***: *p* < 0.001. ^a^ PAG compared with PAC; ^b^ PAI compared with PAG; ^c^ PAI compared with PAC; ^d^ ANOVA test among PAC, PAG, and PAI; ^e^ ADG compared with ADC; ^f^ ADI compared with ADG; ^g^ ADI compared with ADC; ^h^ ANOVA test among ADC, ADG, and ADI.

## Data Availability

The data presented in this study are available on request from the corresponding author.

## Supplementary Materials

The following supporting information can be downloaded at: https://www.mdpi.com/article/10.3390/molecules29071661/s1, Figure S1: PCA (A1,B1) and PLS-DA (A2,B2) score plots based on ^1^H NMR data of serum from children with SS and the corresponding controls; Figure S2: Average ^1^H-NMR spectra of different groups of serum samples; Table S1: The statistical data of the potential biomarkers in serum of children with SS compared with their corresponding controls; Table S2: Identified metabolites from the ^1^H NMR spectra of serum.
