# Supplementary material for: Metabolic Characteristics and Discriminative Diagnosis of Growth Hormone Deficiency and Idiopathic Short Stature in Preadolescents and Adolescents
Source: Molecules. 2024 Apr 7;29(7):1661. doi: 10.3390/molecules29071661 (PMC11013616; doi:10.3390/molecules29071661)
Supplement: Supplementary file 1 [file molecules-29-01661-s001.zip › molecules-2870482-supplementary.pdf]

molecules

MDPI

Supporting Information

Metabolic characteristics and discriminative diagnosis of growth hormone deficiency and idiopathic short stature in pre-adolescents and adolescents

Yajie Chang [1], Jing Chen [2], Hongwei Zhu [3], Rong Huang [2], Jinxia Wu [1], Yanyan Lin [2], Quanquan Li [1], Guiping Shen [1*], Jianghua Feng [1*]

1 Department of Electronic Science, Fujian Provincial Key Laboratory of Plasma and Magnetic Resonance, Xiamen University, Xiamen 361005, China; 1987256610@qq.com (Y.C.), 1262098472@qq.com (J.W.); 2911435264@qq.com (Q.L)

2 Department of Child Health, Women and Children's Hospital, School of Medicine, Xiamen University, Xiamen 361003, China; chenjing8469899@126.com (J.C.); huangrong83@hotmail.com (R.H.); lyy_fjxm@126.com (Y.L.)

3 Education Section and Department of Pediatrics, the First Affiliated Hospital of Bengbu Medical College, Bengbu 233004, China; zhuhongwei51136@126.com (H.Z.)

* Correspondence: gpshen@xmu.edu.cn (G. S.); jianghua.feng@xmu.edu.cn (J. F.)

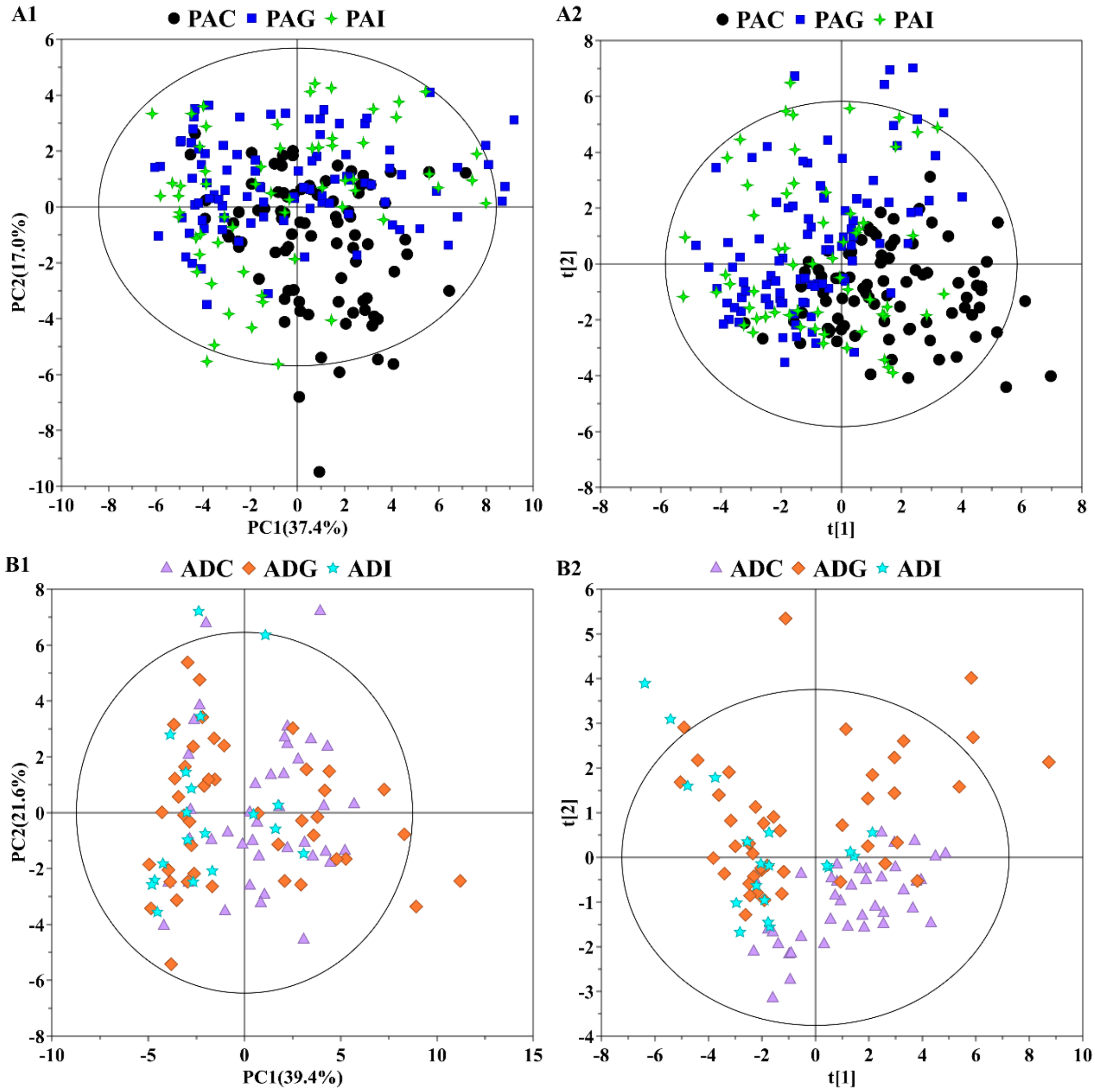

Figure S1. PCA (A1 and B1) and PLS-DA (A2 and B2) scores plots based on 1H NMR data of serum from SS children and the corresponding controls. PAC, preadolescent control group; PAG, preadolescent growth hormone deficiency; PAI, preadolescent idiopathic short stature; ADC, adolescent control group; ADG, adolescent growth hormone deficiency; ADI, adolescent idiopathic short stature

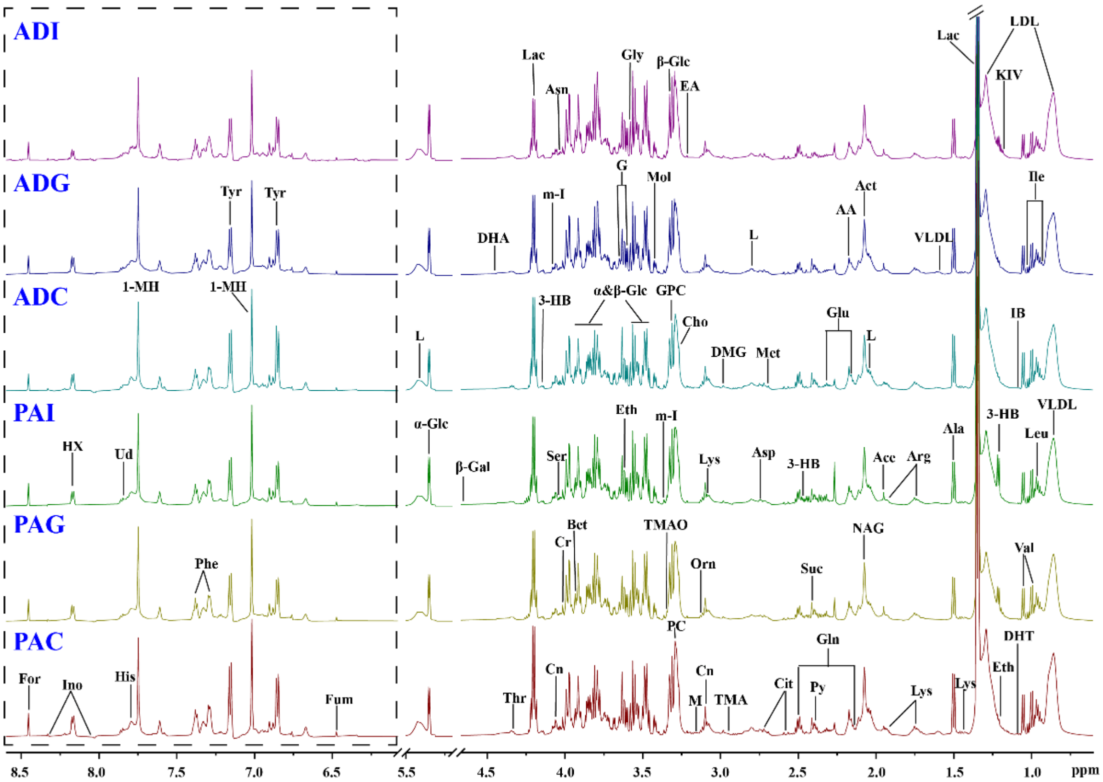

Figure S2. Average ¹H-NMR spectra of different groups of serum samples. The spectral regions in the dashed boxes were vertically expanded 20 times for the purpose of clarity. Keys for the assignments are shown in Table S1 in the supplementary information. PAC, preadolescent control group; PAG, preadolescent growth hormone deficiency; PAI, preadolescent idiopathic short stature; ADC, adolescent control group; ADG, adolescent growth hormone deficiency; ADI, adolescent idiopathic short stature

Table S1 The statistical data of the potential biomarkers in serum of SS children compared with their corresponding controls

	VIP	FC	r	Raw p	Age and sex adjusted *p*	VIP	FC	r	Raw p	Age and Sex adjusted *p*
			**PAG vs. PAC**					**PAI vs. PAC**		
3-HB	3.637	1.487	0.632	2.10E-05	3.78E-08	6.052	1.740	0.727	1.12E-03	3.81E-08
Acetate	1.531	1.285	0.474	2.77E-07	2.08E-14	/	/	/	/	/
Acetoacetate	1.462	1.205	0.519	4.91E-02	5.37E-10	/	/	/	/	/
Acetone	/	/	/	/	/	5.759	1.847	0.681	6.34E-04	4.33E-06
Choline	1.426	1.128	0.559	1.39E-06	4.21E-42	/	/	/	/	/
Ethanol	/	/	/	/	/	1.983	1.057	0.607	2.49E-02	9.76E-45
Glycerol	1.503	1.084	0.553	4.36E-06	4.64E-58	1.721	1.053	0.624	1.25E-02	3.41E-49
Lactate	/	/	/	/	/	8.026	0.878	-0.740	6.97E-03	1.33E-15
Lipid	2.146	0.810	-0.827	8.06E-10	6.17E-31	1.881	0.858	-0.647	1.27E-04	3.91E-20
LDL	6.013	0.959	-0.917	3.98E-02	6.49E-54	/	/	/	/	/
Lysine	1.945	1.032	0.651	4.49E-02	7.82E-62	/	/	/	/	/
NAG	1.860	0.953	-0.720	1.32E-03	4.25E-73	1.605	0.960	-0.603	2.34E-02	2.61E-58
Phosphocholine	1.592	1.085	0.376	4.02E-04	9.67E-45	/	/	/	/	/
Succinate	1.572	1.511	0.661	7.24E-14	2.26E-08	2.576	1.680	0.773	3.84E-09	7.57E-09
VLDL	5.458	0.792	-0.868	3.27E-04	3.21E-10	4.581	0.820	-0.720	4.13E-03	7.33E-05
α-Glucose	3.406	1.185	0.439	4.56E-04	4.51E-10	3.807	1.226	0.501	1.11E-03	6.19E-10
β-Glucose	3.585	1.213	0.470	5.23E-04	3.41E-08	3.909	1.257	0.493	5.82E-04	2.03E-08
			**ADG vs. ADC**					**ADI vs. ADC**		
Acetoacetate	1.474	0.857	-0.598	8.51E-04	1.30E-03	/	/	/	/	/
Alanine	/	/	/	/	/	3.158	0.804	-0.835	1.64E-04	5.00E-04
Asparagine	/	/	/	/	/	1.524	0.917	-0.756	4.97E-02	3.48E-06
Creatine	1.475	0.904	-0.414	1.41E-02	1.33E-04	1.785	0.836	-0.768	2.38E-04	8.10E-06
Creatinine	1.406	0.935	-0.386	2.62E-02	9.31E-06	/	/	/	/	/
Glutamine	2.456	0.805	-0.689	7.69E-08	1.88E-07	2.474	0.879	-0.742	3.24E-03	1.48E-07

Glutamate	/	/	/	/	/	1.917	0.717	-0.863	3.04E-04	9.42E-04
Glycine	/	/	/	/	/	3.154	0.858	-0.674	1.14E-03	9.88E-07
Isoleucine	2.223	0.846	-0.641	2.10E-04	1.29E-04	2.185	0.815	-0.847	2.20E-04	1.59E-02
Lactate	/	/	/	/	/	11.645	0.710	-0.713	5.01E-03	2.01E-03
Leucine	2.414	0.894	-0.529	1.50E-03	1.88E-05	2.582	0.856	-0.851	4.31E-04	2.40E-04
Lysine	2.124	0.927	-0.627	8.22E-03	3.23E-06	1.764	0.906	-0.873	1.11E-02	1.78E-07
Methionine	2.118	0.834	-0.736	3.29E-07	1.62E-09	1.803	0.870	-0.853	4.32E-04	3.43E-08
Ornithine	/	/	/	/	/	2.345	0.867	-0.840	3.52E-03	3.81E-05
Pyruvate	/	/	/	/	/	2.341	0.527	-0.688	9.89E-05	3.02E-02
TMAO	/	/	/	/	/	2.516	0.883	-0.617	2.02E-02	2.69E-07
Valine	3.529	0.863	-0.684	2.16E-04	1.00E-04	3.080	0.798	-0.861	1.36E-05	2.80E-04

Abbreviations: 3-HB, 3-Hydroxybutyrate; LDL, Low Density Lipoprotein; NAG, N-Acetyl-glycoprotein signals; VLDL, Very Low Density Lipoprotein; TMAO, Trimethylamine N-oxide; VIP, Variable importance for projection; FC, Fold change of metabolite ($FC = C_{disease}/C_{control}$). PAC, preadolescent control group; PAG, preadolescent growth hormone deficiency; PAI, preadolescent idiopathic short stature; ADC, adolescent control group; ADG, adolescent growth hormone deficiency; ADI, adolescent idiopathic short stature.

Table S2 Identified metabolites from the ^{1}H NMR spectra of serum

No.	Metabolites	Abbr.	Chemical shift (multiplicity[1])
1	Low density lipoprotein	LDL	0.86(br); 1.28(br)
2	Very Low Density Lipoprotein	VLDL	0.89(br); 1.30(br);1.58(br)
3	Isoleucine	Ile	0.94(t); 1.01(t)
4	Leucine	Leu	0.96(dd)
5	Valine	Val	0.99(d); 1.04(d)
6	Isobutyrate	IB	1.07(d)
7	Dihydrothymine	DHT	1.09(d)
8	α-Ketoisovalerate	KIV	1.14(d)
9	Ethanol	Eth	1.18(t); 3.66(q)
10	3-Hydroxybutyrate	3-HB	1.20(d); 2.31(dd); 2.39(dd); 2.43(dd); 4.16(m)
11	Lactate	Lac	1.33(d); 4.11(q)
12	Lysine	Lys	1.43(m); 1.74(m); 1.89(m); 3.02(t); 3.76(t)
13	Alanine	Ala	1.48(d);
14	Arginine	Arg	1.70(m); 1.90(m); 2.26(t)
15	Acetate	Ace	1.92(s)
16	Lipid	L	2.02(br); 2.23(br); 2.78(br); 5.30(br)
17	N-Acetyl-glycoprotein signals	NAG	2.04(s)
18	Glutamate	Glu	2.08(m); 2.12(m); 2.35(m);
19	Glutamine	Gln	2.12(m); 2.14(m); 2.45(m)
20	Methionine	Met	2.14(s); 2.16(s); 2.65(t)
21	Acetone	Act	2.23(s)
22	Acetoacetate	AA	2.28(s); 3.44(s)
23	Pyruvate	Py	2.37(s)
24	Succinate	Suc	2.41(s);
25	Citrate	Cit	2.53(d); 2.68(d)
26	Aspartate	Asp	2.68(dd); 2.82(dd)
27	Asparagine	Asn	2.85(dd); 2.95(dd); 3.99(m)
28	Trimethylamine	TMA	2.90(s)
29	N,N-Dimethylglycine	DMG	2.93(s)
30	Creatine	Cr	3.04(s); 3.93(s)
31	Creatinine	Cn	3.05(s); 4.06(s)
32	Ornithine	Orn	3.06(t); 3.79(t)
33	Phenylalanine	Phe	3.11(m);7.33(d);7.37(m);7.43(m)
34	Malonate	M	3.11(s)
35	Histidine	His	3.13(m); 7.05(s); 7.77(s)
36	Ethanolamine	EA	3.15(t)
37	Choline	Cho	3.20(s)
38	Phosphocholine	PC	3.21(s)

39	Glycerophosphorylcholine	GPC	3.23(s); 3.35(s); 4.34(m)
40	β-Glucose	β-Glc	3.25(dd); 3.41(t); 3.46(dd); 3.49(t); 3.73(t); 3.90(dd); 4.65(d)
41	Trimethylamine N-oxide	TMAO	3.27(s)
42	*myo*-Inositol	m-I	3.27(t); 3.62(m); 4.07(m)
43	Methanol	Mol	3.36(s)
44	α-Glucose	α-Glc	3.42(t); 3.54(dd); 3.72(t); 3.84(m); 5.24(d)
45	Glycine	Gly	3.56(s)
46	Glycerol	G	3.58(m); 3.66(m)
47	Betaine	Bet	3.90(s)
48	Serine	Ser	3.95(m)
49	Threonine	Thr	4.26(m)
50	Dihydroxyacetone	DHA	4.42(s)
51	β-Galactose	β-Gal	4.52(d)
52	Inosine	Ino	6.10(d); 8.24(s); 8.35(s)
53	Fumarate	Fum	6.52(s)
54	Tyrosine	Tyr	6.90(d); 7.19(d);
55	*para*-Hydroxybenzoate	HBA	6.93(d); 7.81(d)
56	1-Methylhistidine	1-MH	7.03(s); 7.63(s)
57	Uridine	Ud	7.88(s)
58	Hypoxanthine	HX	8.19(s); 8.21(s)
59	Formate	For	8.46(s)

[1] Multiplicity: s, singlet; d, doublet; t, triplet; q, quartet; dd, doublet of doublets; m, multiplet; br, broad resonance.
